# IgG4-related lung disease with multifocal pulmonary consolidations near the pleura: A case report

**DOI:** 10.1097/MD.0000000000030285

**Published:** 2022-08-26

**Authors:** Hitomi Tanaka, Takatoshi Anno, Haruka Takenouchi, Katsumasa Koyama, Hideaki Kaneto, Toru Oga, Yasumasa Monobe, Koichi Tomoda

**Affiliations:** a Department of General Internal Medicine 1, Kawasaki Medical School, Okayama, Japan; b Department of Diabetes, Endocrinology and Metabolism, Kawasaki Medical School, Kurashiki, Japan; c Department of Respiratory Medicine, Kawasaki Medical School, Kurashiki, Japan; d Department of Pathology, Kawasaki Medical School, Okayama, Japan.

**Keywords:** chronic obstructive pulmonary disease, corticosteroid treatment, IgG4-related lung disease, mucinous adenocarcinoma

## Abstract

Immunoglobulin G4 (IgG4)-related disease (IgG4-RD) is a systemic immune-mediated condition that can cause fibroinflammatory lesions in multiple organs. Approximately 35% of IgG4-RD patients have some symptoms in the chest and IgG4-related lung disease (IgG4-RLD) is observed in about 10% of IgG4-RD cases. In addition, it is thought that glucocorticoid therapy is effective for IgG4-RD and IgG4-RLD. It is difficult to diagnose IgG4-RLD complicated with another lung disease.

**Patient concerns:**

An 85-year-old Japanese man was hospitalized due to pulmonary consolidations just below the pleura in chest computed tomography while being treated with antibiotics. Previously, an upper lobectomy of the right lung was performed for an upper lung mucinous adenocarcinoma, and he was diagnosed with chronic obstructive pulmonary disease. Although he took antibiotics before admission, C-reactive protein levels were elevated.

**Diagnosis:**

IgG4 levels were also elevated (IgG4; 733 mg/dL), and lung biopsy histology showed an abundance of IgG4-positive plasma cell infiltration; about 40% of the affected area was occupied by such infiltration. Based on such findings, we finally diagnosed him as IgG4-RLD.

**Interventions:**

We administered 20 mg/d prednisolone.

**Outcomes:**

About 2 weeks after administration of prednisolone by intravenous injection, his multifocal pulmonary consolidations just below the pleura were markedly improved and his pulmonary symptoms disappeared. Four weeks after glucocorticoid therapy, IgG4 levels decreased from 831 mg/dL (peak) to 547 mg/dL.

**Lessons:**

We should consider IgG4-RLD, a rare disease, when lesions are detected as pulmonary consolidations near the pleura and are unresponsive to antibiotic therapy. Glucocorticoid therapy, however, is very effective for such IgG4-RLD.

## 1. Introduction

Immunoglobulin G4 (IgG4)-related disease (IgG4-RD) is a systemic immune-mediated condition that can cause fibroinflammatory lesions in multiple organs and is characterized by fibrosis with prominent lymphoplasmacytic infiltration and dense IgG4-positive plasma cells based on histopathological features.^[1]^ According to previous reports, approximately 35% of IgG4-RD patients have some symptoms in the chest^[2]^ and IgG4-related lung disease (IgG4-RLD) is observed in about 10% of IgG4-RD cases.^[3–5]^ Glucocorticoid therapy is effective for IgG4-RD and IgG4-RLD.

On the other hand, there are few reports of IgG4-RLD complicated with another lung disease such as primary lung cancer,^[6,7]^ inflammatory pseudotumor,^[8]^ and interstitial pneumonia.^[7]^ In addition, the lesion of IgG4-RLD is sometimes observed near the pleura.^[9,10]^ Therefore, it is difficult to diagnose IgG4-RLD in subjects who have another lung disease.

## 2. Case presentation

**Figure 1.**
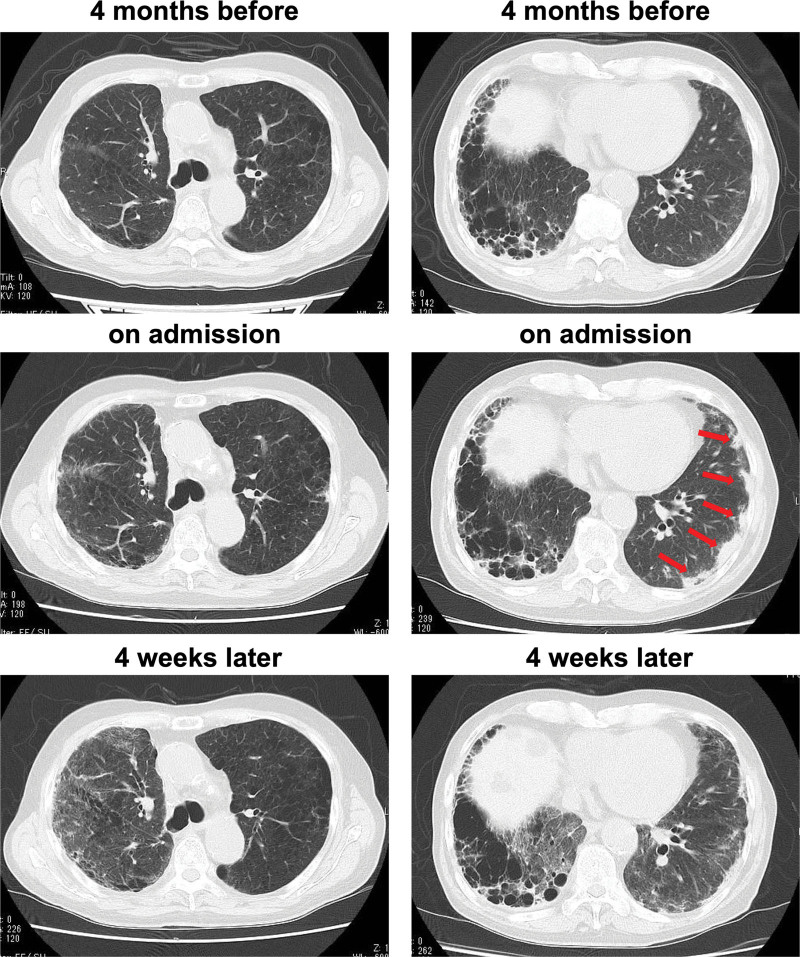
Chest computed tomography (CT) 4 months before (upper panel), on admission (middle panel) and 4 weeks later (lower panel). Chest CT revealed the multifocal pulmonary consolidations just below the pleura (red arrow) on admission, although it was not detected about 4 months before. About 4 weeks after admission (about 2 weeks after injection of prednisolone), his multifocal pulmonary consolidations were improved.

**Figure 2.**
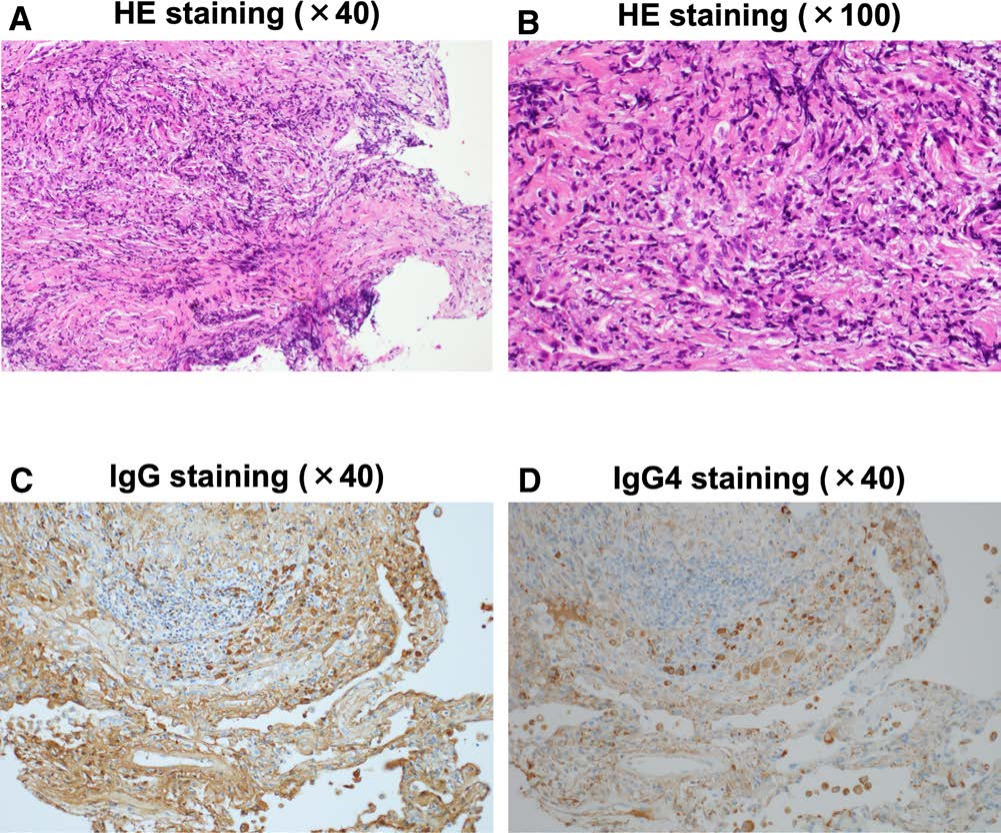
(A and B) HE staining, (C) IgG staining, and (D) IgG4 staining of the lung tissue specimens. There was a marked infiltration of lymphocyte and plasma cells. Histological examination of lung biopsy showed the abundance in IgG4-positive plasma cell infiltration; about 40% of observation area was occupied by such infiltration. IgG4 = immunoglobulin G4.

An 85-year-old Japanese man was hospitalized for the treatment of pulmonary consolidations observed just below the pleura in chest computed tomography (CT), after receiving antibiotics (500 mg of levofloxacin) for 5 days as an outpatient. His history included right upper lung mucinous adenocarcinoma and resulting upper lobectomy of the right lung and a diagnosis of chronic obstructive pulmonary disease (COPD) at age 83. He was a heavy smoker (his total of pack-years = 2 packs/d × 63 years = 126 pack-years) and moderate drinker before experiencing lung mucinous adenocarcinoma after which he stopped both smoking and drinking. His vital signs were as follows: temperature, 36.6°C; blood pressure, 102/54 mm Hg; heart rate, 92 bpm; oxygen saturation, 92% (room air). As shown Table 1, laboratory data on admission were as follows: white blood cell count, 7370/μL (normal range: 3300–8600/μL); red blood cell count, 382 × 10^4^/μL (435–555 × 10^4^/μL); hemoglobin, 11.5 g/dL (13.7–16.8 g/dL); platelet, 23.3 × 10^4^/μL (15.8–34.8 × 10^4^/μL). Liver function was almost within normal range, but renal dysfunction was observed: γ-glutamyl transpeptidase (γ-GTP), 25 U/L (13–64 U/L); alkaline phosphatase (ALP), 304 U/L (106–322 U/L); aspartate aminotransferase (AST), 25 U/L (13-30 U/L); alanine aminotransferase (ALT), 10 U/L (10–42 U/L); creatinine (CRE), 1.65 mg/dL (0.65–1.07 mg/dL); blood urea nitrogen (BUN), 52 mg/dL (8–20 U/L). Although he took antibiotics (500 mg of levofloxacin) before admission, his C-reactive protein (CRP) level was high on admission (CRP, 1.85 mg/dL [<0.14 mg/dL]). We changed the antibiotics to 0.6 g of biapenem. We checked various lung disease markers, because his chest CT revealed multifocal pulmonary consolidations just below the pleura (Fig. 1, middle panel) which was not detected approximately 4 months before (Fig. 1, upper panel). Since he had a history of lung mucinous adenocarcinoma, we discussed the possibility of recurrence of lung carcinoma with some radiologists and decided that it was unlikely. Lung disease-associated data were as follows: immunoglobulin (Ig) G, 3119 mg/dL (861–1747 mg/dL); IgG4, 733 mg/dL (11.0–121.0 mg/dL); IgA, 320 mg/dL (93–393 mg/dL); IgM, 42 mg/dL (33–183 mg/dL); pulmonary surfactant protein-A (SP-A), 79.6 ng/mL (<43.8 ng/mL); SP-D, 216 ng/mL (<110.0 ng/mL), krebs von den lungen-6 (KL-6), 751 U/mL (<500 U/mL). No other autoimmune disease antibodies possibly associated with lung lesion were detected. Therefore, we performed bronchoscopy and transbronchial lung biopsy (TBLB) from right B8, B9 and left B4. Histological examination of TBLB (right B9) showed an abundance of IgG4-positive plasma cell infiltration; with about 40% of the observation area occupied by such infiltration (Fig. 2). Moreover, histological examination revealed lymphoplasmacytic infiltration with associated fibrosis, although there was no obliterative phlebitis. In addition, we evaluated his conditions using the European League Against Rheumatism (EULAR)/American College of Rheumatology (ACR) 2019 Classification^[11]^ and his EULAR/ACR 2019 Classification Score for IgG4-RD was 26 points (13 points of dense lymphocytic infiltrate and storiform fibrosis on histopathology, 6 points of serum IgG4 concentration and 7 points of immunostaining). Based on such findings, we finally diagnosed him as IgG4-RLD. In this case, any other organs were not involved in IgG4-related disease including autoimmune pancreatitis and Mikulicz disease, and there were not any other autoimmune diseases.

**Table 1:** Laboratory data on admission in this subject.

Variable	Result	Reference range	Variable	Result	Reference range
**Peripheral blood**			**Coagulation fibrinolytic system marker**		
White blood cells (/μL)	7370	3300–8600	PT-sec (s)	13.3	9.3–12.5
Neutrocyte (%)	65.8	52.0–80.0	PT-INR	1.20	0.85–1.13
Lymphocytes (%)	22.8	20.0–40.0	PT-activity (%)	73.3	80.7–125.2
Monocyte (%)	7.9	1.0–6.0	APTT (s)	29.2	26.9–38.1
Eosinocyte (%)	2.8	1.0–5.0	Fibrinogen (mg/dL)	389	160–380
Basocyte (%)	0.7	0.0–1.0	**Lung disease marker**		
Red blood cells (×10^4^/μL)	382	435–555	IgG (mg/dL)	3119	861–1747
Hemoglobin (g/dL)	11.5	13.7–16.8	IgA (mg/dL)	320	93–393
Platelets (×10^4^/μL)	23.3	15.8–34.8	IgM (mg/dL)	42	33–183
**Blood biochemistry**			IgG4 (mg/dL)	733	11.0–121.0
Total protein (g/dL)	8.0	6.6–8.1	SP-A (ng/mL)	79.6	<43.8
Albumin (g/dL)	3.2	4.1–5.1	SP-D (ng/mL)	216	<110.0
Globulin (g/dL)	4.8	2.2–3.4	KL-6 (U/mL)	751	<500
Total bilirubin (mg/dL)	0.5	0.4–1.5	**Collagen disease-related antibodies**		
AST (U/L)	25	13–30	Anti-nuclear antibody	9.9 (-)	<20.0
ALT (U/L)	10	10–42	rheumatoid factor (U/L)	<15	0.0–15.0
LDH (U/L)	252	124–222	Anti-ds-DNA Ab. (IU/mL)	<10	0–12
ALP (U/L)	304	106–322	Anti-Sm Ab. (U/mL)	<1.0	<10.0
γ-GTP (U/L)	25	13–64	Anti-SS-A Ab. (U/mL)	<1.0	<10.0
BUN (mg/dL)	52	8–20	Anti-SS-B Ab. (U/mL)	<1.0	<10.0
Creatinine (mg/dL)	1.65	0.65–1.07	Anti-Scl-70 Ab. (U/mL)	<1.0	<10.0
Cholinesterase (U/L)	215	240–486	Anti-Jo-1 Ab. (U/mL)	<1.0	<10.0
Uric acid (mg/dL)	9.3	3.7–7.8	PR3-ANCA (U/mL)	<1.0	<3.5
CRP (mg/dL)	1.85	<0.14	MPO-ANCA (U/mL)	<1.0	<3.5
Plasma glucose (mg/dL)	127		Anti-mitochondrial M2Ab.	<1.5 (-)	<7.0
Hemoglobin A1c (%)	6.0	4.9–6.0	Anti-ARS Ab.	6.6 (-)	<25.0
Total cholesterol (mg/dL)	142	142–248	Anti-MDA5 Ab.	<4 (-)	<32
Sodium (mmol/L)	138	138–145	Anti-CCP Ab. (U/mL)	1.1	<4.5
Potassium (mmol/L)	4.1	3.6–4.8			
Chloride (mmol/L)	107	101–108			

ALP = alkaline phosphatase, ALT = alanine aminotransferase, Anti-aminoacyl tRNA synthetase antibody, Anti-CCP Ab. = Anti-cyclic citrullinated peptide antibody, Anti-ds-DNA Ab. = Anti-double stranded-deoxyribonucleic acid Antibody, Anti-Jo-1 Ab. = PR3-ANCA, proteinase 3 antineutrophil cytoplasmic antibodies, Anti-MDA5 Ab. = Anti-melanoma differentiation-associated gene 5 antibody, Anti-Mitochondrial M2 Ab. = Anti-ARS Ab., Anti-mitochondrial M2 antibody, Anti-Scl-70 Ab. = Anti-Scleroderma-70 antibody, Anti-Sm Ab. = Anti-Smith Antibody, Anti-SS-A Ab. = Anti-Sjögren-syndrome-related antigen A Antibody, Anti-SS-B Ab. = Anti-Sjögren-syndrome-related antigen B Antibody, APTT = activated partial thromboplastin time, AST = aspartate aminotransferase, BUN = blood urea nitrogen, CRP = C-reactive protein, Ig = immunoglobulin, KL-6 = krebs von den lungen-6, LDH = lactate dehydrogenase, MPO-ANCA = myeloperoxidase-antineutrophil cytoplasmic antibodies, PT = prothrombin time, PT-INR = PT-international normalized ratio, SP-A = pulmonary Surfactant Protein-A, SP-D = pulmonary Surfactant Protein-D, γ-GTP = γ-glutamyltranspeptidase.

In general, IgG4-related disease responds well to glucocorticoid therapy. Therefore, we started 20 mg/d of prednisolone. About 2 weeks after administration of prednisolone by intravenous injection, his multifocal pulmonary consolidations just below the pleura were markedly improved (Fig. 1, lower panel) and his pulmonary symptoms disappeared. The pulmonary consolidations were not improved at all despite the prescribed antibiotic therapy (0.6 g of biapenem × 13 days). Four weeks after glucocorticoid therapy, IgG4 levels were decreased from 831 mg/dL (peak) to 547 mg/dL.

## 3. Discussion and Conclusions

Herein, we report a case of IgG4-RLD in a subject with multifocal pulmonary consolidations near the pleura. IgG4-RLD itself is a rare disease. IgG4-RLD is difficult to distinguish from other pulmonary diseases based on histology images alone, and previously there were few reports of IgG4-RLD and IgG4-RLD complicated with another lung diseases.^[6–8]^ Therefore, we think that the present case would provide new and useful information from the clinical point of view.

It is known that subjects with IgG4-RD have various symptoms and show a variety of organ damage to varying degrees. To address the complexity of IgG4-RD, the EULAR/ACR 2019 Classification for IgG4-RD was proposed.^[11]^ This classification is particularly excellent in point that we can distinguish IgG4-RLD from systemic diseases accompanied by lung lesions such as Sjögren syndrome and ANCA (anti-neutrophil cytoplasmic antibody) related diseases. Furthermore, many cases of IgG4-RLD have been reported as non-IgG4-RLD, because of the difficulty of its diagnosis. These results strengthen the idea once more that in diagnosis of IgG4-RLD, it is important to exclude other diseases, and that differential diagnosis is a very difficult task based on symptoms and image inspection. From this point of view, we think that the present case would be worth reporting.

In our patient, at first, we suspected an infectious disease because CRP levels were elevated. However, antibiotic therapy with 500 mg of levofloxacin for 5 days as an outpatient was not effective. In addition, our patient was suspected to have drug-induced pneumonia, because his lung shadows did not change after treatment with antibiotics. Elevated KL-6 concentrations and drug-induced pneumonia suggested complications of interstitial pneumonia at that time. Switching form levofloxacin to 0.6 g of biapenem did not change his pulmonary consolidations. We did not consider that both pulmonary infection and drug-induced pneumonia were causes of pulmonary consolidations. Next, it was important to exclude the possibility of recurrence of mucinous adenocarcinoma. Therefore, we performed bronchoscopy and lung biopsy of pulmonary consolidations just below the pleura. Histological examination of his lung biopsy showed an abundance of IgG4-positive plasma cell infiltration which was compatible with IgG4-RLD feature. On the other hand, he had COPD and he was a heavy smoker before experiencing lung mucinous adenocarcinoma. Since COPD is often complicated with thickness of bronchial walls, it is difficult to distinguish COPD from a bronchovascular type of IgG4-RLD.^[12]^ It is also difficult to differentiate IgG4-RLD from Castleman disease,^[13]^ however, obvious lymph node enlargement was not observed at that time and with the help of pathologists we ruled out Castleman disease. Finally, the direct confirmation of IgG4-positive plasma cell infiltration in the bronchoscopy and lung biopsy of our patient allowed diagnosis of IgG4-RLD, although in general it is difficult to diagnose IgG4-RLD in subjects complicated with another lung disease.

The features of IgG4-RLD lung lesions in chest CT are categorized as solid nodular type, round shaped ground glass opacity type, alveolar interstitial type, and bronchovascular type, with the most typical being bronchovascular type.^[14]^ Our lung findings were multifocal pulmonary consolidations just below the pleura, which were progressive for about 4 months. Fortunately, they were detected during a follow-up screening after upper lobectomy of the right lung. It is rare to experience such lung findings and IgG4-RLD. Therefore, we think that it is important to check IgG4 levels upon observing such multifocal pulmonary consolidations.

Taken together, we should bear in mind the possibility of formation of multifocal pulmonary consolidations near the pleura in subjects with IgG4-RLD. In this case, IgG4-RLD detected as pulmonary consolidations just below the pleura in chest CT was unresponsive to antibiotic therapy. We should know that in such a case there is a possibility of IgG4-RLD and that glucocorticoid therapy is effective for IgG4-RLD lesions.

## Acknowledgment

We thank Dr Askew David (University of Occupational and Environmental Health, Japan) for the English Language editing and review.

## Author contributions

TA is the guarantor of this work and, as such, had full access to all data in the study and takes responsibility for the integrity of the data and the accuracy. HT, TA, HT, KK, and YM researched the data and conducted inspections. HT, TA, HT, KK, and YM were in charge of treatment during hospitalization. HK, TO, NO, and KT reviewed and edited the manuscript. HT, TA, HT, KK, YM, HK, TO, NO, and KT contributed to discussion.

**Conceptualization:** Takatoshi Anno.

**Data curation:** Hitomi Tanaka, Takatoshi Anno, Haruka Takenouchi, Katsumasa Koyama.

**Formal analysis:** Hitomi Tanaka, Takatoshi Anno, Haruka Takenouchi, Katsumasa Koyama.

**Investigation:** Hitomi Tanaka, Takatoshi Anno, Haruka Takenouchi, Katsumasa Koyama.

**Writing – original draft:** Takatoshi Anno, Hideaki Kaneto.

**Writing – review & editing:** Takatoshi Anno, Hideaki Kaneto, Toru Oga, Yasumasa Monobe, Koichi Tomoda.
